# Neonatal extravasation injury: prevention and management in Australia and New Zealand-a survey of current practice

**DOI:** 10.1186/1471-2431-13-34

**Published:** 2013-03-11

**Authors:** Matthew Restieaux, Andrew Maw, Roland Broadbent, Pam Jackson, David Barker, Ben Wheeler

**Affiliations:** 1 Dunedin School of Medicine, University of Otago, Dunedin, New Zealand; 2 Department of Women’s and Child Health, Dunedin School of Medicine, University of Otago, Dunedin, New Zealand

**Keywords:** Neonate, Extravasation, Injury, Hyaluronidase, Intravenous

## Abstract

**Background:**

Extravasation injury remains an important cause of iatrogenic injury in neonatal intensive care. This study aims to describe the current approach to extravasation injury (EI) prevention and management in Neonatal Intensive Care Units (NICUs) in Australia and New Zealand.

**Methods:**

A literature review regarding extravasation injury in the newborn was carried out to inform questionnaire design. An internet-based survey was then conducted with the clinical directors of the 27 tertiary NICUs in Australia and New Zealand.

**Results:**

The survey received a 96% response rate. Approximately two thirds of Australian and New Zealand NICUs have written protocols for prevention and management of extravasation injury. Considerable practice variation was seen for both prevention and treatment of EI. 92% of units had experienced cases of significant EI.

**Conclusions:**

Australian and New Zealand tertiary neonatal units clearly recognise EI as an important cause of iatrogenic morbidity and mortality. Significant variation still exists among units with regards to guidelines for both prevention and management of EI. We recommend that neonatal staff should remain vigilant, ensuring that guidelines for the prevention and treatment of EI are available, and rigorously followed.

## Background

The use of intravenous (IV) access for provision of nutrition and medication is essential in modern neonatal intensive care. Neonatal veins are small and fragile, and intravenous lines are often required for long periods of time. This, in combination with a neonate’s inability to communicate clearly, increases their susceptibility to extravasation injury (EI). EI occurs when fluid from an IV line leaks into the surrounding tissues or other extra-vascular space. Tissue damage occurs as a result of differences in physiochemical characteristics, including pH and osmolarity, between the extravasate substance and the host tissue [1]. Depending on IV line location (central or peripheral), the infiltrate can cause damage potentially resulting in: skin loss, tendon and nerve damage, limb amputation, [2] and central injuries (e.g. hepatic and cardiac). Commonly used neonatal infusions prone to EI include: total parenteral nutrition (TPN), calcium, potassium, bicarbonate, and dextrose in high concentrations. Some particular intravenous medications are also well known for their potential to cause EI such as: acyclovir, vancomycin, and inotropes e.g. dopamine [3].

A number of studies have looked at rates of EI in neonatal intensive care units (NICUs). A 1985 [4] study of 100 NICU survivors 16–29 months following discharge identified 61% as having scars consistent with an EI. A grading system ranging from grade 1 (barely perceptible) to grade 4 (functionally significant) was used to indicate the severity of the scarring. While the vast majority of these cases had only minor scarring, four cases had scarring deemed cosmetically or functionally significant. A more recent study [5] from the United Kingdom found that 38/1000 neonates undergoing neonatal intensive care suffered an EI severe enough to result in skin necrosis. The majority of these injuries were at gestations 26 weeks or less (ranging from 23–35 weeks).

Numerous treatment protocols have been published on the management of EI. The only step common to all is that the intravenous infusion should be stopped immediately. Recent protocols appear to favour the use of hyaluronidase injection plus saline flush in the treatment of EI [2,6-8]. However, there are many other treatment regimes described including the use of various creams, ointments, and occlusive dressings. Specific treatments are also available depending on the infusate e.g. topical nitroglycerin for vasoactive medications [9].

Despite the growing literature in this area, there appears to be little consensus between units and countries on how EI should be prevented and/or treated with much of the available evidence coming from case reports and clinical reviews. No studies have been done looking specifically at current practice across NICUs. With this in mind, we utilised the Australian and New Zealand Neonatal Network (ANZNN), to conduct an internet-based survey exploring current practice in tertiary neonatal units concerning EI prevention and management.

## Methods

27 NICUs providing tertiary care (defined by the ANZNN as providing regular advanced airway support/intensive care) were identified using the 2012 ANZNN directory. This included 21 units in Australia and six in New Zealand. All units were contacted over a 4 week period during June/July, 2012. In the first instance, the Clincal Director/Head of each unit was contacted by email and invited to participate in the survey. This was to ensure the most consistent and authoritative response to questions regarding unit extravasation treatment practices. Informed consent consisted of an explanation of the survey and its purpose given in an email to all potential participants, with consent done via an email link which connected directly to a Survey Monkey™ internet based questionnaire (see Additional file 1).

In eight instances, where there was difficulty in contacting the Director, the Deputy Director or a staff neonatologist was contacted to provide this information. All data was confidentially and automatically stored online at completion of the survey for later analysis.

Survey content was informed by a literature review. This involved an Ovid MEDLINE search for relevant articles published between 1946 and 2012 using the following words or terms: Neonates, extravasation, peripheral cannulae, neonatal intensive care, scars, dopamine, total parenteral nutrition, infiltration, ischaemia, alpha-adrenergic, receptor, complications, therapy, intravenous.

Units were excluded from our survey if they were not classified as providing tertiary level neonatal care within the ANZNN. The two listed neonatal emergency transport services in Australia were also excluded. Ethics approval was provided by the University of Otago ethics committee.

## Results

Responses were obtained from 26/27 units, giving a 96% response rate. Table 1 gives a summary of our findings.

**Table 1:** Summary of main survey findings

**Recognition & prevention of EI:**	
Units with written policy/standard practice	24/26 (92%)
Of these, policy includes:	
Regular nursing observations:	21/24 (88%)
Keeping skin over tip of line visible	18/24 (75%)
Line flush before drug administration	14/24 (58%)
**Treatment of EI:**	
Units with written policy/standard practice	24/26 (92%)
Of these, treatment policy includes:	
Immediate line removal	21/24 (88%)
Limb elevation	15/24 (63%)
Saline washout via small incisions	16/24 (67%)
Hyaluronidase	9/24 (38%)
Warm or cold compress	1/24 (4%)
**Complications of EI:**	
Number of units reporting significant extravasation complication	24/26 (92%)
Central injuries reported:	
Cardiac Tamponade (with associated deaths in 2 units)	13/26 (50%)
Hepatic injury	10/26 (38%)
Peritoneal	8/26 (31%)
Retroperitoneal	3/26 (12%)
Pleural	7/26 (27%)
Peripheral injuries reported:	
Limb endangering	6/26 (23%)

### Prevention

A written policy for the prevention and recognition of EI was used by 69% (18/26) of units. A further 23% (6/26) had no written policy but utilised a standard practice. 8% (2/26) of units had no written policy or standard practice. Broken down by country, 83% (5/6) of the New Zealand units had a written policy, compared to 65% (13/20) of Australian units.

Of units with a written policy or standard practice, these contained: regular recorded nursing observations of the site in 88% (21/24), keeping the skin over the tip of the IV catheter visible in 75% (18/24), and a saline flush before administration of potentially harmful substances in 58% (14/24).

85% (22/26) of units permit the infusion of TPN via peripheral access. In addition, 58% (15/26) of units have concentrated TPN solutions available for exclusive central use (e.g. containing 12.5–15% dextrose). However nine of the units that do infuse TPN peripherally qualified their answer with specific comments such as: “*used reluctantly when no other options available*”, “*prefer and encourage use of central / long line*, *but allow peripheral*”, “*only when swift transition to milk anticipated*”, *and* “*occasionally*, *for short term use when central IV access not available*”.

69% (18/26) of the units allow dopamine/inotropes to be infused via a peripheral line. However ten of these units made additional clarifying comments such as “*short term only whilst central access being obtained*”, and “*if we have no other choice*”. 50% (13/26) of units keep a list of drugs which are particularly likely to cause significant EI.

### Treatment

Regarding the treatment of EI, 65% (17/26) of units have a written policy, with another 27% (7/26) having a standard practice. The remaining 8% (2/26) have neither. Comparing the countries, 83% (5/6) of the New Zealand units have a written policy, compared to 60% (12/26) of Australian units.

To assess severity of EI, 58% (14/24) of units with a written policy or standard practice, use a staging system. One unit, without a formal staging system, uses clinical photographs to monitor severity.

In those with a written policy/standard practice, management included: immediate line removal 88% (21/24) (the remaining units remove the line following specific treatments); elevation of the limb 63% (15/24); a saline washout via small incisions/punctures around the extravasation site 67% (16/24); the use of hyaluronidase 38% (9/24); and a warm or cold compress in 4% (1/24). No units reported current use of a liposuction technique.

If EI occurred with a vasoactive substance (e.g. dopamine or other inotrope), other than the above techniques, 27% (7/26) of units used a specific antidote e.g. Phentolamine or nitroglycerine.

### Complications

Frequency of referral to plastic surgical services was considered. 38% (10/26) of units referred the majority of patients, while 8% (2/26) never requested a plastic surgery referral. Specific comments regarding criteria for review included: “*grade 3 or 4 injury*”; “*early if skin loss looks likely*”; and “*large area of extravasation*, *discolouration*, *and ulceration*”.

Finally, we explored unit experience with severe and/or life threatening complications of central or peripheral EI. 92% (24/26) had experienced a significant extravasation complication. Central injuries reported included: cardiac (tamponade) in 50% (13/26), with associated deaths in 2 units; hepatic injury in 38% (10/26); peritoneal in 31% (8/26); retroperitoneal in 12% (3/26); and pleural in 27% (7/26). Peripheral injuries reported included: limb endangering in 23% (6/26).

## Discussion

Extravasation injury as a complication of neonatal intensive care remains an important cause of iatrogenic morbidity and mortality. 92% of units surveyed reported having experienced a significant extravasation incident. While much of the focus and concern regarding EI centres around peripheral venous access, the occurrence of life-threatening central complications is high, with half the units in this survey reporting experience of cardiac tamponade, with some associated deaths. This highlights the importance of vigilance and monitoring in both central and peripheral IV access.

As the complexity of neonatal care increases, NICUs are increasingly using evidence-based practice protocols. This is particularly important in an environment where monitoring and management is provided by front-line nursing and medical staff at varying levels of training. Our survey reveals that approximately two thirds of Australian and New Zealand NICUs have protocols for the prevention and management of EI; however, as previously noted in the UK, considerable diversity exists between units regarding practice.

It is clear that a number of techniques are being employed for prevention and monitoring of EI. Regarding peripheral IV lines: More than three-quarters of units surveyed reported a policy of regular nursing observations and ensuring IV site visibility, as originally described by Millan [10]. Over half the units have concentrated TPN preparations for exclusive use in central lines, with 15% disallowing peripheral TPN altogether. Based on many qualifying comments received to this question, it is clear most units take a cautious approach to peripheral TPN. The practice of peripheral dopamine infusion is similar, with the majority allowing this with significant qualifications and caution, and some disallowing it altogether. Education and identification of preparations posing risk is clearly an important preventative strategy. 50% of units currently identify and document preparations posing particular risk. Regarding central lines, it is well recognised these carry a risk of potentially serious harm [11]. Nevertheless, it is possible to have a low rate of serious and life-threatening complications with strict adherence to safety criteria [12].

In terms of treatment, while most agree on line removal, subsequent steps vary. Much of this diversity may be explained by the paucity of robust evidence in the literature. 38% currently use hyaluronidase. Hyaluronidase is an enzyme which breaks down constituents of the extracellular matrix, leading to increased diffusion and a subsequent decrease in concentration of the toxic infusate substance [13]. The benefits of this in EI have been shown in animal studies and in a number of case reports in neonates [6,7,13-16]. However, as hyaluronidase is generally used in conjunction with the multiple puncture and saline wash-out technique, it is unclear whether it adds anything over saline alone [17].

Techniques such as multiple skin puncture and saline flush with or without hyaluronidase are invasive, and carry some morbidity. Accurate case selection is vital. Using a staging system (Table 1) is used by over half of the units in this survey, and the practice is referenced in numerous previous papers [1,17,18]. This allows protocols to be devised which guide treatment based on injury severity. For example, stage I and II injuries often do not require treatment, while stage III and IV are likely to require intervention [1,17]. Another strategy is greater involvement of plastic surgical services in decision making around EI. This is now used by over a third of units, for the majority of their EIs. Whatever the method used, with increasing data on the benefits of these or similar treatment techniques, accurate and prompt assessment and treatment is essential (Table 2).

**Table 2:** **Staging of extravasation injuries**[17]**as adapted from Millan**[10]

**Stage**	**Characteristics**
I	Painful intravenous site, no erythema, no swelling
II	Painful intravenous site, slight swelling, no blanching, good pulse below intravenous site, brisk capillary refill below intravenous site
III	Painful intravenous site, marked swelling, blanching, skin cool to touch, good pulse below intravenous site, brisk capillary refill below intravenous site
IV	Painful intravenous site, very marked swelling, blanching, skin cool to touch, decreased or absent pulse, capillary refill over 4 s, skin breakdown or necrosis.

While TPN would be the most implicated agent in EI, dopamine and other catecholamines, widely used as a treatment to support the maintenance of cardiac output and BP, are notorious for causing EI due to their vasoactive properties [3]. The extravasation of dopamine leads to the activation of alpha-adrenoreceptors in the peripheral vasculature, [19] resulting in vasoconstriction and subsequent tissue hypoxia [20]. Various treatment regimes aim to prevent/interrupt this cascade, e.g. phentolamine [9] and nitroglycerin ointment, [21,22]. Phentolamine antagonises alpha-adrenergic receptors, preventing vasoconstriction and subsequent tissue necrosis, [19] while nitroglycerin acts on vascular smooth muscle in arteries and veins, leading to vasodilation and increased perfusion of tissues [21]. Despite 69% of the units administering vasoactive substances (e.g. dopamine) by peripheral venous access, only 25% use a specific antidote, such as phentolamine or nitroglycerin ointment, in the treatment of catecholamine-induced EI.

This is the first survey investigating prevention and management of extravasation injury in Australian and New Zealand neonatal intensive care units. Responses were obtained from senior clinicians with close to 100% response rate, ensuring that this survey provides an accurate and reliable picture of the current practice.

## Conclusions

Australian and New Zealand tertiary neonatal units clearly recognise EI as an important cause of iatrogenic morbidity and mortality. Significant variation still exists among units with regards to guidelines for both prevention and management of EI. Pending further research to inform best practice in this area, neonatal staff should remain vigilant, ensuring that guidelines for the prevention and treatment of EI are available, and conscientiously followed.

## Competing interests

The authors declare no competing interests in this project.

## Authors’ contributions

MR, AM and BW conducted the survey. All authors were involved in survey design, interpretation of results and editing the final manuscript. MR and AM wrote the first draft of the manuscript. BW conceived the study, participated in its design and coordination and helped to draft/edit the manuscript. All authors read and approved the final manuscript.

## Pre-publication history

The pre-publication history for this paper can be accessed here:

http://www.biomedcentral.com/1471-2431/13/34/prepub

## Supplementary Material

Additional file 1 Survey. Click here for file
