# Supplementary material for: Neonatal extravasation injury: prevention and management in Australia and New Zealand-a survey of current practice
Source: BMC Pediatr. 2013 Mar 11;13:34. doi: 10.1186/1471-2431-13-34 (PMC3599986; doi:10.1186/1471-2431-13-34)
Supplement: Additional file 1 — Survey. [file 1471-2431-13-34-S1.pdf]

Extravasation injuries in the neonatal population

This survey is aimed at describing current extravasation policy in Australian and New Zealand NICUs. All level 3 and 2A units will be surveyed.

Only one reply is required for each NICU and in the first instance the Medical Director (according to ANZNN) of each NICU has been emailed with this survey.

The survey takes less than 5 minutes to complete.

The first question requests the name of the unit so that we can follow up any nonrespondents, or clarify any particular details. The information regarding individual units will remain confidential.

*1. From which unit are you reporting? (name and city)

2. Does your unit have a written policy for the prevention and early recognition of extravasation injuries?

- Yes, written policy
- No written policy but have a standard practice
- No written policy or standard practice
- Don't know

3. For units that have a written policy or standard practice, which of these options does it contain? (select as many as apply)

- Regular recorded nursing observations
- Keeping the skin over the tip of the IV catheter visible
- A saline flush before administration of potentially harmful substances
- Don't know

Other (please specify)

4. Does your unit infuse TPN via peripheral access?

- Yes
- No

Comment

5. Does your unit have routine TPN solutions for exclusive use in central lines? (e.g. TPN containing 12.5% dextrose for use in central access only)

- ○ Yes
- ○ No

Comment

6. Does your unit have a list of drugs which are particuarly likely to cause serious problems on extravasation?

- ○ Yes
- ○ No

Comment

7. Does your unit have a written protocol for the treatment of extravasation injuries?

- ○ Yes, written policy
- ○ No written policy but have a standard practice
- ○ No written policy and no standard practice
- ○ Don't know

8. For those with a written protocol or standard practice, does it contain a staging system for severity of injury?

- ○ Yes
- ○ No

If no, how are the severity of extravasation injuries classified

9. For those with a written protocol or standard practice for extravasation injuries involving TPN or similar non-vasoactive fluid, which of these does your management guideline contain? (select as many as apply)

- ☐ Remove the line
- ☐ Elevate the affected limb
- ☐ Saline washout with small incisions around the extravasation site
- ☐ Hyaluronidase
- ☐ Liposuction
- ☐ Warm or cold compress

Other (please specify)

10. Is it permissible in your unit to infuse dopamine via a peripheral line?

- Yes
- No
- Don't know

Comment

11. For extravasation with a vasoactive substance (e.g. inotrope) does your treatment differ from that above? (e.g. do you use a specific antidote such as Phentolamine or nitroglycerine?)

- Yes
- No
- Don't know

12. For what percentage of extravasation injuries requiring treatment would your unit request plastic surgical review?

- All of the time
- 90% or more of the time
- 50% or more of the time
- Less than 50% of the time
- Less than 10% of the time
- Not at all

Under what particular circumstances would you obtain Plastics review?

13. Has your unit had any significant central extravasation injuries/ complications?

- Yes
- No
- Don't know

14. If yes, please tick as many as apply

- ☐ Cardiac (e.g. Tamponade)
- ☐ Hepatic
- ☐ Peritoneal
- ☐ Retroperitoneal
- ☐ Pleural
- ☐ Limb endangering

Other (please specify)

15. Regarding complications: To the best of your knowledge, has your unit had a baby requiring amputation as a result of extravasation in the past 15 years?

- ○ Yes
- ○ No
- ○ Don't Know

Please comment if needed

16. Regarding complications: To the best of your knowledge, has your unit had a death associated with extravasation in the past 15 years? - e.g. secondary to cardiac tamponade / hepatic injury

- ○ Yes
- ○ No
- ○ Don't Know

If yes – what was the injury leading to death? E.g. Cardiac tamponade/ Hepatic injury; and was this related to a PICC or Umbilical catheter

Thank you for your time and effort in filling out this survey. We will distribute the results of the survey to all units when available.
